# Environmental context predicts state fluctuations in negative symptoms in youth at clinical high risk for psychosis

**DOI:** 10.1017/S0033291723001393

**Published:** 2023-12

**Authors:** Lauren Luther, Ian M. Raugh, Delaney E. Collins, Alysia Berglund, Anna R. Knippenberg, Vijay A. Mittal, Elaine F. Walker, Gregory P. Strauss

**Affiliations:** 1 Department of Psychology, University of Georgia, Athens, GA, USA; 2 Department of Psychology, Northwestern University, Evanston, IL, USA; 3 Department of Psychology, Emory University, Atlanta, GA, USA

**Keywords:** Anhedonia, asociality, avolition, clinical-high risk, environment, psychosis

## Abstract

**Background:**

Negative symptoms (avolition, anhedonia, asociality) are a prevalent symptom in those across the psychosis-spectrum and also occur at subclinical levels in the general population. Recent work has begun to examine how environmental contexts (e.g. locations) influence negative symptoms. However, limited work has evaluated how environments may contribute to negative symptoms among youth at clinical high risk for psychosis (CHR). The current study uses Ecological Momentary Assessment to assess how four environmental contexts (locations, activities, social interactions, social interaction method) impact state fluctuations in negative symptoms in CHR and healthy control (CN) participants.

**Methods:**

CHR youth (*n* = 116) and CN (*n* = 61) completed 8 daily surveys for 6 days assessing negative symptoms and contexts.

**Results:**

Mixed-effects modeling demonstrated that negative symptoms largely varied across contexts in both groups. CHR participants had higher negative symptoms than CN participants in most contexts, but groups had similar symptom reductions during recreational activities and phone call interactions. Among CHR participants, negative symptoms were elevated in several contexts, including studying/working, commuting, eating, running errands, and being at home.

**Conclusions:**

Results demonstrate that negative symptoms dynamically change across some contexts in CHR participants. Negative symptoms were more intact in some contexts, while other contexts, notably some used to promote functional recovery, may exacerbate negative symptoms in CHR. Findings suggest that environmental factors should be considered when understanding state fluctuations in negative symptoms among those at CHR participants.

## Introduction

Negative symptoms, including avolition, anhedonia, and asociality, are commonly observed in psychotic disorders and are also present at subclinical levels in healthy controls (Bobes, Arango, Garcia-Garcia, & Rejas, 2010; Stefanis et al., 2002). Recently, there has been increased interest in understanding processes contributing to negative symptoms in youth at clinical high-risk for psychosis (CHR) due to their clinical and prognostic significance. Negative symptoms are highly prevalent among CHR youth, with 82% of participants in the North American Prodrome Longitudinal Study reporting one or more negative symptom (Piskulic et al., 2012). Further, negative symptoms are associated with social and role functioning impairments (Glenthøj, Kristensen, Wenneberg, Hjorthøj, & Nordentoft, 2020; Schlosser et al., 2015), are one of the earliest markers of psychosis risk that lead to first contact with the treatment system (Yung & McGorry, 1996), and are significant predictors of transition to a full psychotic disorder (Healey et al., 2018; Zhang et al., 2020). Thus, determining processes contributing to negative symptoms in CHR youth may allow early identification and prevention efforts to shift to the earliest phase of the prodrome when treatments may have maximum potential to prevent or delay illness onset.

Prior mechanistic research has largely focused on determining whether negative symptom models developed in psychosis-spectrum disorders apply to CHR youth. These studies have examined the integrity of reward-processing components that rely on cortico-striatal circuitry and whether associated behavioral or neural abnormalities predict negative symptom severity (Kring & Barch, 2014; Strauss, Waltz, & Gold, 2014). Much like psychotic disorder samples, CHR youth demonstrate an association between negative symptoms and abnormalities in effort-cost computation (Strauss, Bartolomeo, & Luther, 2021), reinforcement learning (Karcher, Hua, & Kerns, 2019; Strauss et al., 2021; Waltz et al., 2015), value representation (Bartolomeo, Chapman, Raugh, & Strauss, 2021), and reward anticipation (Millman et al., 2020). However, reward-processing differences have also been observed between psychotic disorder and CHR youth samples. For example, hedonic reactivity (i.e. self-reported or neurophysiological response to pleasant stimuli or rewards) is generally thought to be intact in psychotic disorders (Cohen & Minor, 2010; Strauss et al., 2014). However, several laboratory-based studies have demonstrated that hedonic capacity (or consummatory pleasure) is reduced in CHR youth (Gruber, Strauss, Dombrecht, & Mittal, 2018; Schlosser et al., 2014; Strauss, Ruiz, Visser, Crespo, & Dickinson, 2018; Yee et al., 2019), suggesting CHR youth may have more of a true hedonic deficit than those with psychotic disorders.

In addition to person-level processes like reward-processing, there are likely other mechanisms underlying negative symptoms. The Bioecosystem Theory of Negative Symptoms (Strauss, 2021) proposes that environmental factors interact with person-level, biological, cognitive, and psychological factors to play an important role in the onset and maintenance of negative symptoms across phases of psychotic illness. This theory proposes that direct and indirect environmental contexts have a significant influence on negative symptoms. The immediate environment, or the microsystem (e.g. locations including work or people one interacts with), is theorized to directly influence negative symptoms levels. In support of this theory, we recently found that negative symptoms levels varied across different activities (e.g. resting *v.* recreational, school/work activities), locations (e.g. home *v.* school/work), social partners (e.g. alone *v.* family, friends), and methods of social interactions (i.e. social modality; alone *v.* electronic communication) in schizophrenia (SZ) (Luther, Raugh, Collins, Knippenberg, & Strauss, 2023). Indirect environmental factors that reduce access to resources for recreational, goal-directed, and social activities (e.g. street walkability) have also been associated with greater negative symptoms in SZ (Zhang, James, & Strauss, 2022). Collectively, these studies suggest that the environment plays a critical, yet previously underestimated role in negative symptoms in those with a psychotic disorder.

To our knowledge, no studies have examined how environmental contexts influence negative symptoms in CHR youth. Examining how context may influence negative symptoms in this population could identify contexts that may be critical for optimizing negative symptom prevention efforts or early interventions. Indeed, environments likely differ between CHR youth and SZ, with CHR youth engaging in a wider range of contexts and activities. For example, CHR youth spend a greater amount of time online, including interacting with others electronically, than healthy controls (CN) (Mittal, Tessner, & Walker, 2007; Pelletier-Baldelli, Strauss, Visser, & Mittal, 2017). In addition, CHR youth may be more connected to their microsystems, including living with family, attending secondary education, and connecting with a wider range of social contacts than SZ. Thus, research is needed to evaluate how these environments may uniquely impact negative symptoms in CHR youth. However, clinical ratings scales, the most frequently used method to assess negative symptoms, were largely designed for adults with SZ, often have a singular rating that conflates symptom levels across contexts, and do not capture the range of activities youth engage in.

This study leveraged Ecological Momentary Assessment (EMA) to examine the impact of dynamic contexts on temporally specific negative symptoms in CHR youth. EMA offers a sensitive, naturalistic approach for examining the wide range of activities that youth are engaged in throughout daily life. Although a few studies have successfully used EMA in CHR youth, almost all have focused on positive symptoms and emotional experience (e.g. positive affect) (Michel et al., 2022; Paetzold et al., 2021; Palmier-Claus, Taylor, Gooding, Dunn, & Lewis, 2012; van der Steen et al., 2017). Thus, we used EMA to examine the role that activity (e.g. recreation, computer use), location (e.g. home, public), social partner (e.g. friend), and social modality (e.g. electronically, in-person) had on temporally related negative symptoms of avolition, anhedonia, and asociality in CHR youth and CN. In line with our prior findings in SZ (Luther et al., 2023), we hypothesized that negative symptoms in CHR youth would change dynamically across different contexts. Given that CHR youth may be characterized by a true hedonic deficit more so than SZ (i.e. based on laboratory-based studies showing that CHR but not SZ show reductions in hedonic capacity; Cohen & Minor, 2010; Gruber et al., 2018; Strauss et al., 2018), we also hypothesized that anhedonia would demonstrate the fewest changes across contexts compared to avolition and asociality and would be more consistently reduced in CHR youth compared to CN.

## Methods

### Participants

Participants were 61 CN (2080 EMA samples) and 116 CHR youth (3658 EMA samples). CHR participants were recruited from programs at the University of Georgia, Northwestern University, and Emory University that are designed to perform evaluations for youth displaying psychotic experiences. CN were recruited from the local community using printed and online advertisements. Study procedures were approved by the local institutional review board.

CHR participants met criteria for a psychosis-risk syndrome on the Structured Interview for Psychosis-Risk Syndromes (SIPS; Miller et al., 2003) and did not meet Structured Clinical Interview for DSM-5 (SCID-5; First, Williams, Karg, & Spitzer, 2015) lifetime psychotic disorder criteria. CN did not meet SCID-5 criteria for any current psychiatric disorder. See the supplement for CHR syndrome types and additional recruitment and eligibility criteria. CHR and CN did not significantly differ in personal education, parental education, sex, or race; however, CHR were trending toward being older than CN (*p* = 0.05). CHR also completed fewer EMA surveys than CN (see Table 1); 3.28% (*n* = 2) of CN and 16.5% (*n* = 20) of CHR participants completed fewer than 25% of surveys. To maximize available data, we did not exclude any participants based on adherence, consistent with more recent recommendations and the robustness of mixed-effects models for missing data (Hoffman & Rovine, 2007; Schielzeth et al., 2020).

**Table 1.** Demographic and clinical characteristics

	CHR (*n* = 116)	CN (*n* = 61)	Test statistic χ^2^/*F*	*p* value
Age	22.27 (4.09)	21.10 (2.71)	4.04	0.05
Parental education	15.30 (3.00)	15.88 (2.73)	1.54	0.22
Participant education	13.87 (2.71)	14.34 (1.43)	1.57	0.21
Male; *n* (%)	26 (22.4%)	13 (21.3%)	0.03	0.87
Race; (*n*, %)			5.39	0.37
African American	17 (14.7%)	5 (8.2%)		
Caucasian	60 (51.7%)	41 (67.2%)		
Asian American	16 (13.8%)	8 (13.1%)		
Hispanic/Latino	12 (10.3%)	3 (4.9%)		
Biracial	10 (8.6%)	3 (4.9%)		
Other	1 (0.9%)	1 (1.6%)		
EMA Survey Adherence; *M* (SD)	53.4% (26.6%)	70.9% (23.1%)	19.16	<0.001

CHR, Clinical high-risk group; CN, healthy control group.

*Note.* Means and standard deviations (in parentheses) presented unless otherwise noted.

### Procedures

Study procedures were completed across one week. Procedures were similar to past SZ studies (Luther et al., 2023; Raugh et al., 2021).

*Initial Laboratory Visit*. After written informed consent, all participants completed the SCID-5. CHR also completed the SIPS. After diagnostic consensus, participants downloaded the mEMA smartphone app (ilumivu.com), received training on using the app, and completed a practice EMA survey to ensure they understood the response formats.

*EMA Surveys.* EMA surveys were delivered via the mEMA app for 6 days. From 9AM to 9PM, participants were randomly notified within 90-minute epochs about surveys 8 times per day. This survey amount was chosen to capture changes in context and negative symptoms. Once notified, participants had 15 min to complete surveys. Surveys took less than five minutes and utilized skip logic to minimize time burden. Negative symptom items measured the internal experience components of anhedonia, avolition, and asociality via questions about enjoyment, interest, and motivation for their concurrently endorsed activity and social context (see supplement for items). Context items assessed current activity, location, social partner, and social modality. Contexts were not mutually exclusive; participants selected as many contexts that they engaged in within a 15-min window prior to the survey.

*Return to Laboratory.* Following EMA, participants were compensated $30 per hour of interviews and $1 per EMA survey completed, with a bonus of up to $60 for completing ⩾5 surveys per day and >80% of surveys.

### Data analysis

Following prior analytic methods (Luther et al., 2023), all models presented used mixed-effects modeling with random intercepts within person and day in order to account for nesting and repeated measures in the data. All effects evaluated used categorical variables; therefore, no random slopes were used. Models were conducted separately for each negative symptom domain (anhedonia, avolition, asociality) and context (activity, location, social partner, social modality). Contexts observed less than 200 times out of 3212 total surveys were excluded from analyses. Of the remaining contexts, eight activity contexts were evaluated (resting, working/studying, watching TV, eating, computer use, errands, recreation/hobbies, commuting). In addition, four location contexts (home, school/work, public, friend/family residence), six social partner contexts (no one/alone, family, significant other, coworkers/classmates, friends, strangers) and four social modality contexts [no one/alone, in-person, electronic (text, social media, etc.), phone/video call] were examined.

Primary models for each negative symptom domain measured the effect of Group (CN *v.* CHR), Context (see list above), and Group × Context interaction (see supplement for a post-hoc power analysis). Context was treated as a repeated measure within each survey instance. To isolate the relative effects of each context (i.e. does context B reduce symptoms compared to context A), only instances where activities were endorsed were included in the primary models. Model omnibus effects were evaluated using Wald χ^2^ tests, and significant main effects of Context were followed by post-hoc contrasts to assess differences between Contexts. Significant Group × Context interactions indicate that the difference in negative symptoms among the groups varies by context. Thus, for significant Group × Context interactions, both between-group and within-group effects were decomposed. Specifically, post-hoc tests evaluated how the two groups differed from each other at each context (e.g. when at work, how did CHR differ from CN on negative symptoms) and how contexts differed from each other within the two groups (e.g. how were negative symptoms different when engaging with strangers compared to being alone in CN and CHR). A False Discovery Rate correction (Benjamini & Hochberg, 1995) was also applied to each term (Group, Context, Group × Context) across models to control for multiple comparisons. Cohen's d accounting for unequal group sizes (Cohen, 1988) was used to determine group difference effect sizes within each context. To determine effect sizes for differences between contexts within each group, Cohen's d average (Cohen, 1988) was used to account for the repeated-measure nature of the effects.

Several exploratory models were planned and presented in online Supplementary materials to better contextualize the primary model results, including: (1) group differences and COVID differences (before *v.* during) in Context endorsement (e.g. likelihood each group endorses a Context), (2) effects in overall negative symptoms (i.e. sum of anhedonia, avolition, and asociality), (3) differences in anticipatory and consummatory pleasure anhedonia based on the effect of Group, Context, and Group × Context interactions, and (4) the influence of hedonic and goal-directed activity composite contexts on negative symptoms. Finally, given the high rate of mood disorders in CHR (Kline et al., 2018), we examined the association between the presence of a mood disorder and negative symptoms to see if mood disorder status should be controlled for in analyses. Analyses were conducted using R version 4.2.2, and analytic code can be downloaded through a link in the online Supplementary materials.

## Results

Broadly, groups did not significantly differ in their probability of endorsing different contexts. However, CHR were more likely to be resting or having phone call interactions than CN (online Supplementary Table S1). Mood disorder status was not significantly related to negative symptoms (online Supplementary Table S2) and thus was not controlled for in remaining analyses.

**Figure 1.**
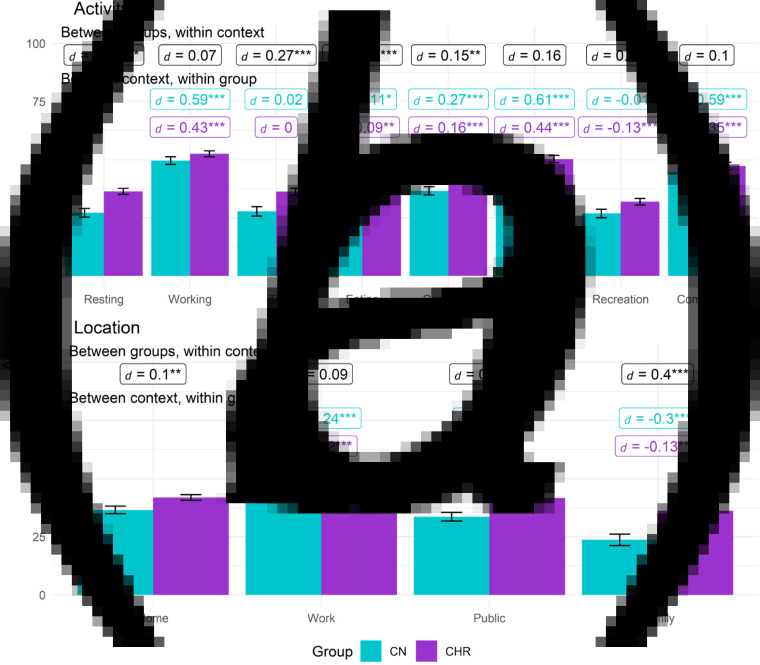
Anhedonia by group and activity or location contexts. (*a*) activity. (*b*) Location. *Note.* Between groups, within context labels reflect contrast between groups within each context while between context, within group labels reflect contrast within group relative to reference context (e.g. resting or being at home). Figures use estimated marginal means and error bars reflect standard error. Work contexts also included school (e.g. item was school/work), while family location also includes friends. * = *p* < 0.05, ** = *p* < 0.01, ** = *p* < 0.001.

**Figure 2.**
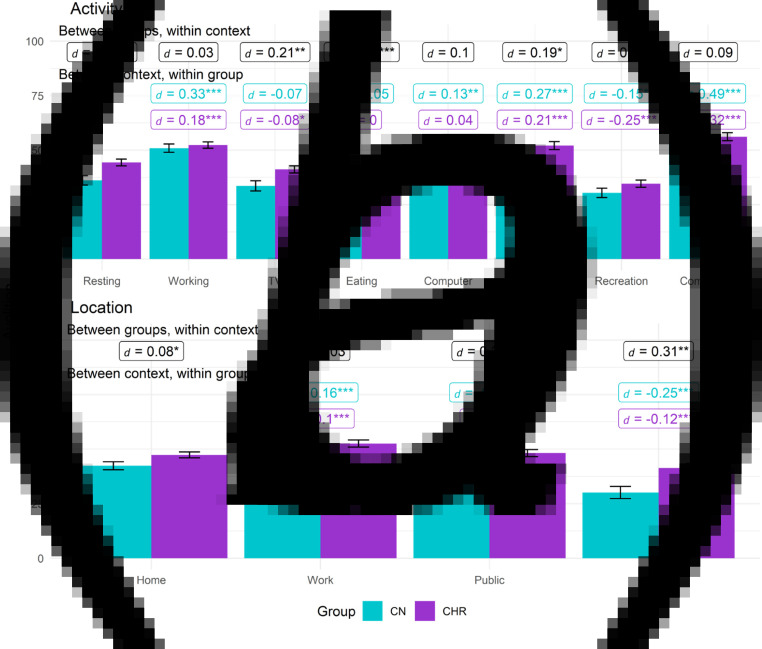
Avolition by group based on activity and location contexts. (*a*) activity. (*b*) Location. *Note.* Between groups, within context labels reflect contrast between groups within each context while between context, within group labels reflect contrast within group relative to reference context (resting or at home). Figures use estimated marginal means and error bars reflect standard error. Work contexts also included school (e.g. item was school/work), while family location also includes friends. * = *p* < 0.05, ** = *p* < 0.01, ** = *p* < 0.001.

**Figure 3.**
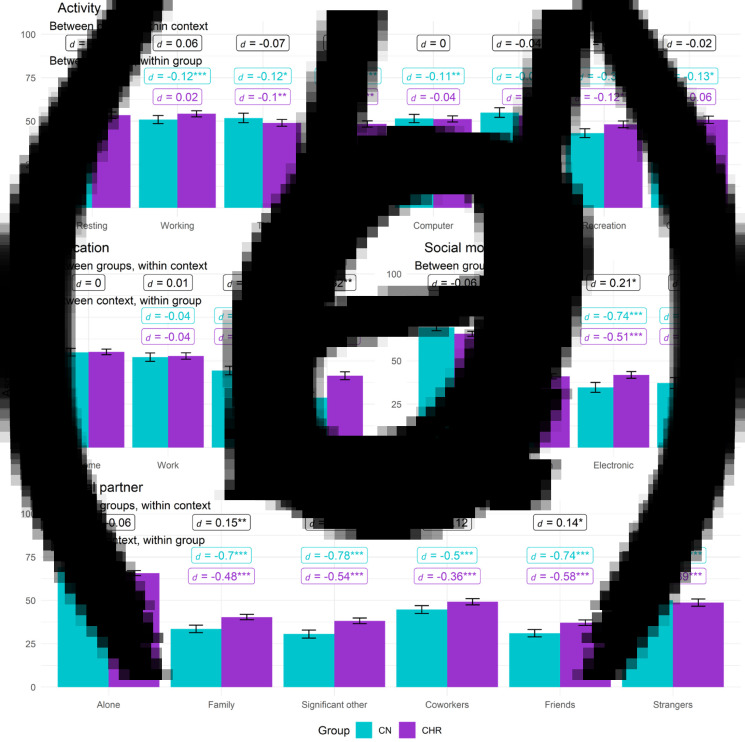
Asociality by group for activity, location, social partner, and social modality. (*a*) activity. (*b*) Location. (*c*) social modality. (d) social partner. *Note.* Between groups, within context labels reflect contrast between groups within each context while between context, within group labels reflect contrast within group relative to reference context (resting, being at home, or being alone). Figures use estimated marginal means and error bars reflect standard error. Work contexts also included school (e.g. item was school/work), family location also includes friends, and coworker social partners also included classmates. * = *p* < 0.05, ** = *p* < 0.01, ** = *p* < 0.001.

Table 2 contains omnibus effects for models examining the effects of context on anhedonia, avolition, and asociality. When significant, two-way interactions between Group and Context are shown in Figs 1–3.

**Table 2.** Omnibus model effects

Context	Total *R*^2^	Fixed-effect *R*^2^	Group	Context	Group × Context
Anhedonia					
Activity	0.44	0.1	16.3***	533.85***	24.72***
Location	0.36	0.05	7.08**	152.6***	12.29**
Social partner	0.39	0.06	4.7*	214.44***	8.91
Social modality	0.35	0.03	3.77	72.64***	5.97
Avolition					
Activity	0.4	0.06	9.58**	283.08***	27.53***
Location	0.32	0.03	4.73*	93.37***	23.11***
Social partner	0.34	0.03	4.88*	73.45***	5.13
Social modality	0.31	0.02	4.41*	22.17***	4.07
Asociality					
Activity	0.46	0.01	1.31	89.49***	27.3***
Location	0.41	0.03	0	130.16***	16.49***
Social partner	0.52	0.22	2.1	1171.27***	57.06***
Social modality	0.49	0.2	2.37	955.84***	45.14***

*Note*. Effects in bold remained after a False Discovery Rate Correction. The Fixed-effect *R*^2^ is the variance accounted for by the fixed effects, while the Total *R*^2^ includes the variance accounted for by both the fixed effects and the random effects.

## Anhedonia

### Activity

For activity, the Group, Context, and Group × Context effects were all significant. CHR displayed greater anhedonia than CN while resting, watching TV, eating, using the computer, and during recreation, while no group differences were found for studying/working, errands, or commuting. In both CHR and CN, compared to when resting, anhedonia was greater when studying/working, eating, using the computer, running errands, or commuting; however, compared to resting, anhedonia was significantly lower during recreational activities only in CHR. Anhedonia was lowest during recreation compared to other activities in CHR (*t*s > 3.35, *p*s < 0.001, *d*s > 0.13).

### Location

For location, the Group, Context, and Group × Context effects were all significant. CHR had higher anhedonia than CN in all locations except school/work. Within both groups, compared to being at home, anhedonia was higher while at school/work and lower when at a friend or family member's home. Compared to being at home, CN but not CHR reported lower anhedonia when in public. For both groups, anhedonia was highest while at school/work (*t*s > 7.5, *p*s < 0.001, *d*s > 0.23).

### Social partner

For social partner, Group and Context effects were significant, while the Group × Context was nonsignificant. Across all social partners, CHR endorsed greater anhedonia than CN (*d* = 0.08). In the combined sample, compared to being alone, anhedonia was lower when with family (*t* = 10.33, *p* < 0.001, *d* = 0.18), significant other(s) (*t* = 12.57, *p* < 0.001, *d* = 0.26), and friends (*t* = 14.91, *p* < 0.001, *d* = 0.27), but higher when with classmates or coworkers (*t* = 3.3, *p* = 0.001, *d* = 0.07).

### Social modality

For social modality, the main effect of Context was significant, while the Group and Group × Context effects were nonsignificant. In the combined sample, relative to being alone, anhedonia was lower during in person (*t* = 11.25, *p* ⩽ 0.001, *d* = 0.15), electronic (*t* = 5.06, *p* ⩽ 0.001, *d* = 0.14), and phone call (*t* = 2.56, *p* = 0.011, *d* = 0.08) interactions.

### Anticipatory and consummatory anhedonia

Both anticipatory and consummatory effects were broadly consistent with the overall anhedonia analyses (online Supplementary Table S4 and Figures S2–S4).

## Avolition

### Activity

For activity, the Group, Context, and Group × Context effects were all significant. Compared to CN, avolition was greater in CHR while resting, watching TV, eating, and running errands; groups did not differ on avolition during studying/working, computer, recreation, or commuting activities. Within each group, compared to being at rest, avolition was higher during studying/working, running errands, and commuting but lower during recreational activities. Within CHR but not CN, compared to being at rest, avolition was lower when watching TV. When compared to being at rest, CN but not CHR also showed greater avolition when on the computer. Within CHR, avolition was lowest during recreation compared to other contexts (*t*s > 4, *p*s < 0.001, *d*s > 0.17).

### Location

For location, the Group, Context, and Group × Context effects were all significant. Compared to CN, CHR demonstrated greater avolition at home, in public, and at a friend or family member's home; no group differences emerged for school/work. Compared to being at home, both groups reported greater avolition when at school/work and lower avolition when at a friend/family member's home. In CN but not CHR, compared to being at home, avolition was lower when in public than when at home. In both groups, avolition was greatest while at school/work (*t*s > 2.8, *p*s < 0.005, *d*s > 0.1).

### Social partner

For social partner, Group and Context effects were significant, while Group × Context was nonsignificant. Across all social partners, CHR endorsed greater avolition than CN (*d* = 0.06). In the combined sample, relative to being alone, avolition was greater when around classmates/coworkers (*t* = 2.5, *p* = 0.012, *d* = 0.05) but lower in all other contexts (*t*s > 2.7, *p*s < 0.006, *d*s > 0.07).

### Social modality

For social modality, Group and Context effects were significant, while Group × Context was nonsignificant. Across all social modalities, avolition was greater among CHR compared to CN (*d* = 0.08). In the combined sample, compared to being alone, avolition was significantly lower for in person (*t* = 6.35, *p* < 0.001, *d* = 0.08), electronic (*t* = 1.96, *p* = 0.05, *d* = 0.05), and phone call interactions (*t* = 2.18, *p* = 0.029, *d* = 0.06).

## Asociality

### Activity

For activity context, Context and Group × Context effects were significant, while Group was nonsignificant. However, post-hoc comparisons revealed groups did not significantly differ on asociality in any activity context. Among CN, asociality was significantly higher when at rest compared to all other activity contexts except errands. Among CHR, compared to being at rest, asociality was lower when watching TV, eating, or engaging in recreation but did not significantly differ from rest for school/work, errands, computer, or commuting activities.

### Location

For location context, Context and Group × Context effects were significant, while Group was nonsignificant. CHR displayed higher asociality than CN while at a friend/family home; groups did not differ on asociality in other locations. In both groups, compared to being at home, asociality was lower when in public or at a friend/family member's house.

### Social partner

For social partner, Context and Group × Context effects were significant, while Group was nonsignificant. Compared to CN, asociality was greater among CHR when interacting with family, a significant other, or friends. In both groups, relative to being alone, asociality was lower in all social partner contexts.

### Social modality

For social modality, Context and Group × Context effects were significant, while Group was nonsignificant. CHR showed greater asociality than CN during in person and electronic but not phone call interactions. In both groups, compared to being alone, asociality was lower during in person, electronic, and phone call interactions, and no significant differences in asociality levels were present in either group between in-person, electronic, and phone call interactions (*t*s < −1.29, *p*s < 0.20, *d*s < 0.04).

## Discussion

Given the prognostic significance of negative symptoms in CHR youth (Piskulic et al., 2012), there is a critical need to identify potentially modifiable mechanisms such as one's immediate environmental context that contribute to negative symptoms. This study used EMA to provide a more naturalistic assessment of whether environmental contexts lead to changes in state negative symptoms among CHR youth. Building on the limited work that has used EMA to examine negative symptoms in CHR youth, our results suggest that across both CHR and CN, anhedonia, avolition, and asociality levels largely varied across activity, location, social partner, and social modality contexts in daily life. These results align with the Bioecosystem Model of Negative symptoms (Strauss, 2021) and indicate that the immediate environment or microsystem can lead to dynamic changes in negative symptoms. These results also build on other models of psychosis risk and symptom development such as the Stress Diathesis Model of Schizophrenia (Nuechterlein & Dawson, 1984; Walker & Diforio, 1997) and the Cognitive Model of Negative Symptoms (Beck, Rector, Stolar, & Grant, 2009) which implicate early and later life environmental factors such as social adversity and urbanization in the development of psychosis and associated symptoms in vulnerable individuals (Dean & Murray, 2005).

Fluctuations in negative symptoms across contexts were especially evident for avolition and asociality. For avolition, we found significant within-group differences in CHR youth, indicating that compared to being at rest, being at school/work, running errands, and commuting were associated with greater avolition, while engaging in recreation and watching TV were linked to lower avolition. Thus, much like SZ (Luther et al., 2023), activities more commonly associated with being ‘productive’ were associated with increases in avolition, while more pleasure-based activities were linked to reduced avolition. Given evidence of a hedonic deficit in CHR youth (e.g. Gruber et al., 2018), this may suggest that increasing engagement in more pleasure-based activities to improve the experience of pleasure could overtime lead to improved avolition and engagement in productive activities. Higher-stakes performance-based activities (e.g. school, work) may also activate defeatist beliefs about performance (Clay, Raugh, Bartolomeo, & Strauss, 2021), resulting in greater avolition. In line with this, recreational activities led to similar avolition reductions for both groups (i.e. non-significant group differences). This aligns with our prior results in SZ (Luther et al., 2023), suggesting that recreation and community engagement may be especially critical for inclusion in avolition interventions across the psychosis-spectrum. Finally, there was some evidence that locations differentially impacted avolition in CHR youth. Among CHR, compared to being at home, being at a family/friend's house was associated with avolition reductions, while being at school/work was associated with greater avolition. Somewhat surprisingly, being at home and being in public led to similar avolition levels in CHR participants, while being in public (*v.* home) led to lower avolition in CN. This may be due to positive symptom exacerbations (e.g. paranoia) that may occur when CHR youth are in novel or busy public places; future work is needed to identify which types of public locations are most linked with symptom exacerbations as well as the impact of more personalized and meaningful locations on concurrent and prospective negative symptoms.

As expected, asociality fluctuated mostly between social and non-social contexts in both groups. Although no group differences were observed in asociality or interest in interactions across different activity types, significant group differences emerged between social partner, social modality, and location contexts. Notably, among social partner types, CHR participants reported less social interest in close relationships (family, significant others, friends) than CN, while no group differences emerged for more distal relationships (coworkers/classmates, strangers). This aligns with prior work showing that being with friends was associated with increased psychosis-proneness scores and anxiety (Husky, Grondin, & Swendsen, 2004). Close relationships may be more challenging for CHR youth, potentially as a result of subthreshold symptoms (e.g. paranoia, social cognition reductions) that could exacerbate developmentally normative conversational challenges that can arise during youth. However, despite these group differences, engaging in these close relationships was still associated with asociality reductions when compared to being alone in CHR youth. Asociality was also the only domain where groups differed on symptom levels across the different interaction methods. This was driven by the increased asociality observed in CHR compared to CN participants when interacting in person and electronically. However, there were no group differences for phone calls, and all interaction methods were associated with lower asociality in CHR compared to being alone. Groups also did not differ on anhedonia or avolition levels across the different interaction methods. Thus, despite spending more time interacting with others online than CN (Mittal et al., 2007; Pelletier-Baldelli et al., 2017), CHR youth appear to benefit from a similar reduction in negative symptoms during both electronic-based and in person interactions. Taken together, this work suggests that online or social media interventions (e.g. Alvarez-Jimenez et al., 2021) could be useful for targeting negative symptoms in CHR youth.

While anhedonia also appeared to change across different contexts within both groups, in accordance with hypotheses, there was also evidence for greater anhedonia elevations in the CHR group. Compared to CN, CHR participants reported less pleasure (i.e. greater anhedonia) when engaging in most activities (except school/work, errands, commuting) and across all social partners and most locations (except school/work). This contrasts our prior work showing that SZ and CN reported similar anhedonia levels across most contexts (Luther et al., 2023). However, these results in CHR youth further support prior work with self-report measures and laboratory paradigms demonstrating that CHR youth experience more of a true hedonic deficit than SZ (e.g. Gruber et al., 2018). This finding was further supported when we examined consummatory and anticipatory anhedonia; CHR participants reported greater reductions in both in-the-moment and anticipatory pleasure compared to CN across most contexts and negative symptom domains. However, there were a few contexts where anhedonia was significantly reduced in CHR participants compared to other contexts. This included recreational activities and being at a family/friend's home. Thus, there may still be a few areas where anhedonia is more preserved in CHR youth, which could be bolstered in early intervention and prevention efforts.

Several limitations should be considered. First, although a study strength was the large CHR sample, we could not evaluate effects based on transition to a psychotic disorder due to lack of a longitudinal follow-up. Second, additional work is needed to clarify the direction of the observed effects and examine how context at a preceding point influences subsequent negative symptoms (and vice versa). This study also focused only on contexts that are part of the immediate environment (e.g. the microsystem); future studies could examine how indirect environmental factors (e.g. street walkability) dynamically interact with immediate environmental contexts to impact symptoms. Further, CHR participants had lower EMA adherence than CN; although there was no apparent systematic effect of this difference on results, this possibility cannot be definitively ruled out. Relatedly, although steps were taken to increase survey accessibility (e.g. surveys were available for 15 min), participants may have missed the survey completion window due to engagement in an activity or social interaction. Additionally, we could not examine how expressive negative symptoms (e.g. blunted affect) are impacted by environmental contexts or how socioeconomic status may influence the role of context on negative symptoms. Although the naturalistic assessment of activities in daily life is a study strength, some youth (in both groups) may engage in more prescribed activities (e.g. attending high school, greater family engagement), which could impact results. Finally, although supplemental analyses did not indicate significant differences in the interpretation of the effects of context on negative symptoms for those who completed the study prior *v.* after COVID, we cannot rule out the possibility that restrictions imposed during the pandemic impacted other aspects of negative symptoms.

Despite these limitations, several clinical implications can be drawn from these findings. Among CHR for psychosis youth, environmental context may directly influence symptom levels of anhedonia, avolition, and asociality. Further, there are some contexts where negative symptoms may be more preserved among CHR youth. These results can guide prevention and early intervention efforts by identifying specific contexts clinicians can guide participants to engage in (e.g. recreation, being with friends or family), reduce time in (e.g. being at home), or modify (e.g. work, commuting, running errands, being in public) to improve and potentially prevent the onset of clinically significant negative symptoms. These results may also guide novel mobile health interventions by identifying specific contexts where negative symptom exacerbations may occur and therefore where in-the-moment intervention content may be most needed and impactful.

## Supporting information

Luther et al. supplementary material Luther et al. supplementary material

## Supplementary material

The supplementary material for this article can be found at https://doi.org/10.1017/S0033291723001393.
