# Supplementary material for: Environmental context predicts state fluctuations in negative symptoms in youth at clinical high risk for psychosis
Source: Psychol Med. 2023 May 29;53(16):7609–18. doi: 10.1017/S0033291723001393 (PMC10755225; doi:10.1017/S0033291723001393)
Supplement: Luther et al. supplementary material [file S0033291723001393sup001.docx]

**SUPPLEMENTAL MATERIALS FOR**

ENVIRONMENTAL CONTEXT PREDICTS STATE FLUCTUATIONS IN NEGATIVE SYMPTOMS IN YOUTH AT CLINICAL HIGH RISK FOR PSYCHOSIS

Lauren Luther^1^*

Ian M. Raugh^1^*

Delaney Collins^1^

Alysia Berglund^1^

Anna R. Knippenberg^1^

Vijay A. Mittal^2^

Elaine F. Walker^3^

Gregory P. Strauss^1^

*Equal Contribution

1. Department of Psychology, University of Georgia, Athens, GA, USA
2. Department of Psychology, Northwestern University, Evanston, IL, USA
3. Department of Psychology, Emory University, Atlanta, GA, USA

Corresponding authors:

Gregory P. Strauss, Ph.D., Email: gstrauss@uga.edu. Phone: +1-706-542-0307. Fax: +1-706-542-3275. University of Georgia, Department of Psychology, 125 Baldwin St., Athens, GA 30602; Lauren Luther, Ph.D., Email: [lauren.luther@uga.edu](mailto:lauren.luther@uga.edu). Phone: +1-706-542-2174. Fax: +1-706-542-3275. University of Georgia, Department of Psychology, 125 Baldwin St., Athens, GA 30602

Contents

[Analytic code 4](#_Toc128333971)

[Additional Recruitment, Participant, and Eligibility Information 5](#_Toc128333972)

[EMA Questions for Context and Negative Symptoms 6](#_Toc128333973)

[Additional Results 8](#_Toc128333974)

[Power Analysis 8](#_Toc128333975)

[Mood Disorder Status and Association with Negative Symptoms 8](#_Toc128333976)

[Overall negative symptoms 8](#_Toc128333977)

[Anticipatory and consummatory anhedonia 9](#_Toc128333978)

[Supplemental Table 1. Context frequency by group and COVID status (during or before) 11](#_Toc128333979)

[Supplemental Table 2. Association between Mood Disorder Status and EMA avolition, anhedonia, and asociality 13](#_Toc128333980)

[Supplemental Table 3. Overall Negative Symptoms omnibus effects 14](#_Toc128333981)

[Supplemental Table 4. Anticipatory and consummatory pleasure omnibus models 15](#_Toc128333982)

[Supplemental Table 5. Omnibus model effects for Goal-Directed and Hedonic Activity Composites 16](#_Toc128333983)

[Supplemental Table 6. Effects of preceding negative symptoms on context probability 17](#_Toc128333984)

[Figures 19](#_Toc128333985)

[Supplemental Figure 1. Overall Negative Symptoms 19](#_Toc128333986)

[Supplemental Figure 2. Anticipatory and consummatory pleasure - Activity 20](#_Toc128333987)

[Supplemental Figure 3. Anticipatory and consummatory pleasure - Location 21](#_Toc128333988)

[Supplemental Figure 4. Anticipatory and consummatory pleasure - Social 22](#_Toc128333989)

[Supplemental Figure 5. Hedonic and Goal-Directed Activities – Anhedonia 23](#_Toc128333990)

[Supplemental Figure 6. Hedonic and Goal-Directed Activities – Avolition 24](#_Toc128333991)

[Supplemental Figure 7. Hedonic and Goal-Directed Activities – Asociality 25](#_Toc128333992)

[Supplemental Figure 8. Hedonic and Goal-Directed Activities – Overall Negative Symptoms 26](#_Toc128333993)

[References 27](#_Toc128333994)

## Analytic code

Due to the length of the code (> 10,000 lines or 600 pages), code is available for download on OSF: <https://osf.io/xpd2u>

## Additional Recruitment, Participant, and Eligibility Information

**Recruitment Sites**

Clinical-High Risk for Psychosis (CHR) participants were recruited from one of three sites: the Georgia Psychiatric Risk Evaluation Program (G-PREP) at the University of Georgia in Athens, GA, the Northwestern University Adolescent Development and Preventative Treatment (ADAPT) research program in Evanston, IL, or the Mental Health and Development (MHAD) Program at Emory University in Atlanta, GA. These programs perform evaluations for youth displaying psychotic experiences.

**CHR Syndromes**

One-hundred and thirteen CHR participants met criteria for Attenuated Positive Symptoms Syndrome (APSS; i.e., SIPS score of 3–5 on ≥ one positive symptom item); 63 met criteria for APSS progression, 48 for APSS persistence, and 2 for APSS partial remission. Three participants met criteria for multiple syndromes: APSS Persistence and Brief Intermittent Psychotic Syndrome (BIPS) full remission (*n* = 1); APSS persistence, BIPS full remission, and Genetic Risk and Deterioration Syndrome (GRD) full remission (*n* = 1); APSS and GRD Progression (*n* = 1).

**Additional Eligibility Criteria**

CN also did not have a lifetime psychotic disorder history or have a family psychosis history. No participants had a lifetime neurological disease.

## EMA Questions for Context and Negative Symptoms

- 1. Where are you? **[Location context]**
     1. Home
     2. Work/ School
     3. Friend/ Family home
     4. Public place (bus, store, etc)
     5. Treatment/ Doctor’s office
  2. What are you doing? **[Activity Context]**
     1. Working/ Studying
     2. Eating/ Drinking
     3. Recreation/ Hobby
     4. Internet/ Computer use
     5. Errands/ Housework
     6. Resting
     7. Exercising
     8. Shopping
     9. TV/ Music
     10. Smoking
     11. Commuting/ Traveling
     12. Bathing/ Hygiene
     13. Pacing restlessly
     14. Socializing
     15. Nothing *(Proceed to 1.4)*
  3. Please complete the following:
     1. How interested are you in the activity? **[Avolition item]**
     2. How much are you enjoying the activity? **[Anhedonia – consummatory]**
     3. How much do you think you will enjoy that activity the next time you do it? **[Anhedonia – anticipatory]**
  4. *(If 1.2.15 selected)* How much do you want to be doing an activity right now?^C^
  5. Who are you interacting with? **[Social Partner Context]**
     1. Significant other
     2. Family/ Roommates
     3. Friends
     4. Coworkers/ Classmates
     5. Doctor/ Therapist
     6. Strangers
     7. No one/ Alone *(Proceed to 1.8)*
  6. How are you interacting with them? **[Social Modality Context]**
     1. In person
     2. Phone/ Video call
     3. Electronically (text, social media, etc.)
  7. Please complete the following:
     1. How interested are you in this social interaction? **[Asociality Item]**
     2. How much are you enjoying this social interaction? **[Anhedonia – consummatory]**
     3. How much do you think you will enjoy interacting with them next time? **[Anhedonia – anticipatory]**
  8. *(If 1.5.7 selected)* How much do you want to be interacting with someone right now?

## Additional Results

### [Power Analysis](#_Additional_Results)

Post-hoc power analyses were conducted using a summary-statistics approach (Murayama et al., 2022). Between-subjects effects of d ≥ 0.45 were adequately powered (i.e., 80% power or greater). Within-subjects effects (i.e., context) of d ≥ 0.21 were adequately powered while effects of 0.15 were powered at 50%.

### Mood Disorder Status and Association with Negative Symptoms

Of those with CHR, 44 had a current mood disorder diagnosis based on the SCID and 78 CHR participants had a lifetime mood disorder diagnosis. We examined the association between the presence of a mood disorder and summary-level EMA anhedonia, avolition, and asociality. None of the associations were significant between current or lifetime mood disorder status and anhedonia, avolition, and asociality (*r*s < .17) (see Supplemental Table 2). Thus, given that mood disorder status did not seem to have a strong impact on negative symptoms in this sample, we did not account for mood disorder status in the remaining analyses.

### Overall negative symptoms

See supplemental Table 3 and supplemental Figure 1.

**Activity.** There were significant Group, Context, and Group X Context effects on overall negative symptoms. Compared to CN, overall negative symptoms were greater in CHR when at rest, eating, and during recreation. Within both groups, compared to resting, overall negative symptoms were greater when studying/working, using the computer, running errands, or commuting but lower when engaging in recreation. Relative to being at rest, CHR but not CN showed reduced symptoms when watching TV, while CN but not CHR showed reduced negative symptoms while eating.

**Location.** There were significant Context and Group X Context effects on overall negative symptoms for location; the effect of Group was nonsignificant. Compared to CN, CHR demonstrated greater overall negative symptoms in public or at a friend/family home. In both groups, compared to being at home, overall negative symptoms were greater when at work/school but lower when in public or at a friend/family home.

**Social partner.** The effects of Context and Group X Context on overall negative symptoms for social partner were significant, while the effect of Group was nonsignificant. Compared to CN, CHR endorsed greater overall negative symptoms when interacting with family, a significant other, or friends compared to CN. Among both groups, compared to being alone, overall negative symptoms were lower with any interaction partner.

**Social modality.** The effects of Context and Group X Context on overall negative symptoms for social modality were significant, while the effect of Group was nonsignificant. Overall negative symptoms did not significantly differ between CHR and CN when alone; however, compared to CN, CHR showed greater overall negative symptoms across every other interaction modality. Further, both groups showed reductions in overall negative symptoms when not alone.

### Anticipatory and consummatory anhedonia

See Supplemental Table 4 and Supplemental Figures 2-4.

**Anticipatory – activity**. The effects of Group, Context, and Group X Context on anticipatory anhedonia for activity context were significant. Compared to CN, CHR had higher anticipatory anhedonia while resting, watching tv, eating, going on the computer, and during recreation. Within CHR, compared to resting, anticipatory anhedonia was significantly higher when studying/working, eating, going on the computer, running errands, or commuting, while it was lower when engaged in recreational activity.

**Anticipatory – location.** The effects of Group, Context, and Group X Context on anticipatory anhedonia for location were significant. Compared to CN, SZ had significantly anticipatory anhedonia while at home, in public, or with family. Within CHR, compared to be being at home, anticipatory anhedonia was significantly higher when at school/work, while it was lower when with family.

**Anticipatory – social partner.** The effect of Context on anticipatory anhedonia for social partner was significant, while the effects of Group and Group X Context were nonsignificant. In the combined sample, compared to being alone, being with family, significant other, or friends was linked with lower anticipatory anhedonia, while it was higher with co-workers/classmates.

**Anticipatory – social modality.** The effect of Context on anticipatory anhedonia for social modality was significant, while the effects of Group and Group X Context were nonsignificant. In the combined sample, relative to being alone, anticipatory anhedonia was lower during in person, electronic, and phone-video call interactions.

**Consummatory – activity.** The effects of Group, Context, and Group X Context on Consummatory anhedonia for activity context were significant. Compared to CN, CHR had higher consummatory anhedonia while resting, watching tv, eating, going on the computer, and during recreation. Within CHR, compared to resting, anticipatory anhedonia was significantly higher when studying/working, eating, going on the computer, running errands, or commuting, while it was lower when engaged in recreational activity.

**Consummatory – location.** The effects of Group, Context, and Group X Context on Consummatory anhedonia for location context were significant. Compared to CN, SZ had significantly higher anticipatory anhedonia while at home, in public, or with family. Within CHR, compared be being at home, anticipatory anhedonia was significantly higher when at school/work, while it was lower when with family.

**Consummatory – social partner.** The effects of Group and Context on Consummatory anhedonia for social partner were significant, while the Group X Context interaction was nonsignificant. Across all social partners, CHR reported greater consummatory anhedonia than CN.

**Consummatory – social modality.** The effects of Group and Context on Consummatory anhedonia for social modality were significant, while the Group X Context interaction was nonsignificant. CHR reported higher consummatory anhedonia across all social modality contexts. In the full sample, compared to being alone, consummatory anhedonia was lower during in person, electronic, and phone-video call interactions.

***Hedonic and Goal-Directed Activity Composites***

Two composite activity contexts were produced to encapsulate goal-directed versus hedonic activity. The goal-directed composite included engagement in at least one of the following activities: studying/working, errands, exercising, shopping, or commuting. The hedonic composite included engagement in at least one of the following activities: bathing, computer use, eating, recreation, resting, smoking, or TV. Absolute effects of the goal-directed and hedonic activity composites on negative symptoms were evaluated independently by domain using the effects of Group, Composite (present or absent), and Group X Composite interactions.

See Supplemental Table 5 and Supplemental Figures 5-8 for full results.

**Anhedonia - Goal-directed Activity**. There were significant Group, Goal-directed activity, and Group X Goal-directed activity effects on anhedonia. Anhedonia significantly differed between the groups in the absence of goal-directed activity (t = 4.23, p < .001, d = 0.17); however, this difference was nonsignificant during goal-directed activities (t = 1.96, p = .051, d = 0.078). Anhedonia increased during goal-directed activities in CN (t = 19.65, p < .001, d = 0.4) and CHR (t = 19.46, p < .001, d = 0.32).

**Anhedonia - Hedonic Activity.** There were also significant Hedonic activity and Group X Hedonic activity effects on anhedonia; however, the Group effect was nonsignificant. This interaction was such that anhedonia decreased significantly in both groups during hedonic activities (CN: t = 19.23, p < .001, d = 0.41; CHR: t = 18.04, p < .001, d = .33). Further, while the groups did not significantly differ in anhedonia outside of hedonic activities (t = 1.58, p = .116, d = 0.08), CHR endorsed greater anhedonia during hedonic activities (t = 3.95, p < .001, d = 0.13).

**Avolition - Goal-directed Activity**. The Group, Goal-directed activity, and Group X Goal-directed activity effects on avolition were all significant. In the absence of goal-directed activity, CHR were significantly higher than CN on avolition (t = 3.55, p < .001, d = 0.14); however, they did not significantly differ during goal-directed activities (t = 1.72, p = .086, d = 0.07). Avolition increased during goal-directed activities in CN (t = 10.79, p < .001, d = 0.22) and CHR (t = 9.41, p < .001, d = 0.15).

**Avolition - Hedonic Activity.** In the model evaluating Hedonic activity, the effects of Group and Hedonic activity were significant, while the Group X Hedonic activity interaction was nonsignificant. CHR endorsed greater avolition regardless of hedonic activity (d = 0.08). Avolition was lower during hedonic activities (d = 0.29) in both groups.

**Asociality - Goal-directed Activity**. There were no significant effects of Group, Goal-directed activity, nor a Group X Goal-directed activity composite for asociality.

While the Group and Group X Hedonic activity effects were also nonsignificant for asociality, there was a significant effect of Hedonic activity such that asociality was lower during hedonic activities in both groups (d = 0.07).

**Overall negative symptoms - Goal-directed Activity**. The effects of Group, Goal-directed activity, and the Group X Goal-directed activity interaction on overall negative symptoms were all significant. When not engaging in a goal-directed activity, CHR showed significantly greater negative symptoms than CN; however, this was nonsignificant during goal-directed activity. In both groups, negative symptoms were greater during goal-directed activities.

**Overall negative symptoms - Hedonic Activity.** However, the model of hedonic activities only found a significant effect of Hedonic activity with nonsignificant Group and Group X Hedonic activity effects. Overall negative symptoms were lower during hedonic activity.

### Supplemental Table 1. Context frequency by group and COVID status (during or before)

|  | Group | | | COVID | | |
| --- | --- | --- | --- | --- | --- | --- |
| Context | CHR | CN | OR | During | Before | OR |
| Activity |  |  |  |  |  |  |
| Goal-directed | 1661 (45.41%) | 1090 (52.4%) | 0.72  [0.52, 1] | 1483 (44.98%) | 1268 (51.95%) | 0.76  [0.55, 1.06] |
| Hedonic | 2480 (67.8%) | 1309 (62.93%) | 1.3  [0.91, 1.84] | 2236 (67.82%) | 1553 (63.62%) | 1.06  [0.75, 1.49] |
| Resting | 705 (19.27%) | 305 (14.66%) | 1.71**  [1.18, 2.49] | 541 (16.41%) | 469 (19.21%) | 0.69*  [0.48, 0.99] |
| Studying/ Working | 1034 (28.27%) | 694 (33.37%) | 0.66  [0.43, 1.02] | 912 (27.66%) | 816 (33.43%) | 0.77  [0.5, 1.19] |
| TV | 522 (14.27%) | 227 (10.91%) | 1.58  [0.88, 2.84] | 344 (10.43%) | 405 (16.59%) | 0.52*  [0.29, 0.91] |
| Eating | 847 (23.15%) | 417 (20.05%) | 0.96  [0.68, 1.37] | 726 (22.02%) | 538 (22.04%) | 0.85  [0.6, 1.2] |
| Computer | 1000 (27.34%) | 452 (21.73%) | 1.33  [0.8, 2.19] | 874 (26.51%) | 578 (23.68%) | 1.2  [0.74, 1.97] |
| Errands | 321 (8.78%) | 200 (9.62%) | 0.99  [0.64, 1.55] | 318 (9.65%) | 203 (8.32%) | 1.19  [0.76, 1.85] |
| Recreation | 481 (13.15%) | 345 (16.59%) | 0.77  [0.5, 1.17] | 478 (14.5%) | 348 (14.26%) | 1.02  [0.67, 1.55] |
| Commuting | 303 (8.28%) | 144 (6.92%) | 1.31  [0.78, 2.23] | 194 (5.88%) | 253 (10.36%) | 0.6  [0.36, 1.01] |
| Location |  |  |  |  |  |  |
| Home | 2222 (60.74%) | 1150 (55.29%) | 1.17  [0.73, 1.89] | 2151 (65.24%) | 1221 (50.02%) | 2.35***  [1.47, 3.76] |
| School/ Work | 756 (20.67%) | 519 (24.95%) | 0.49  [0.24, 1.02] | 659 (19.99%) | 616 (25.24%) | 0.93  [0.46, 1.9] |
| Public | 663 (18.12%) | 395 (18.99%) | 1.01  [0.69, 1.48] | 482 (14.62%) | 576 (23.6%) | 0.45***  [0.31, 0.66] |
| Friend or family home | 318 (8.69%) | 168 (8.08%) | 1.28  [0.54, 3.03] | 310 (9.4%) | 176 (7.21%) | 1.32  [0.56, 3.09] |
| Social Partner |  |  |  |  |  |  |
| No one/ Alone | 1626 (44.45%) | 944 (45.38%) | 0.98  [0.61, 1.58] | 1579 (47.89%) | 991 (40.6%) | 1.62*  [1.01, 2.58] |
| Family | 904 (24.71%) | 414 (19.9%) | 0.95  [0.55, 1.64] | 836 (25.36%) | 482 (19.75%) | 1.23  [0.72, 2.11] |
| Significant other | 745 (20.37%) | 295 (14.18%) | 2.08  [0.56, 7.81] | 599 (18.17%) | 441 (18.07%) | 1.64  [0.45, 5.98] |
| Classmates/ Coworkers | 383 (10.47%) | 279 (13.41%) | 0.73  [0.39, 1.38] | 284 (8.61%) | 378 (15.49%) | 0.4**  [0.21, 0.74] |
| Friends | 653 (17.85%) | 447 (21.49%) | 0.79  [0.47, 1.32] | 494 (14.98%) | 606 (24.83%) | 0.43**  [0.26, 0.71] |
| Strangers | 207 (5.66%) | 115 (5.53%) | 1.17  [0.56, 2.45] | 134 (4.06%) | 188 (7.7%) | 0.45*  [0.22, 0.93] |
| Social Modality |  |  |  |  |  |  |
| In-person | 1878 (51.34%) | 1099 (52.84%) | 0.8  [0.5, 1.25] | 1597 (48.44%) | 1380 (56.53%) | 0.56*  [0.36, 0.87] |
| Electronic (text, social media, etc) | 307 (8.39%) | 130 (6.25%) | 2  [0.81, 4.97] | 211 (6.4%) | 226 (9.26%) | 0.47  [0.19, 1.12] |
| Phone/ Video call | 265 (7.24%) | 84 (4.04%) | 1.87*  [1.05, 3.31] | 229 (6.95%) | 120 (4.92%) | 1.7  [0.97, 2.98] |

*Note*. OR = Odds Ratio, odds are coded such that values greater than one indicate that a context was more likely to occur in CHR or during COVID compared to CN or before COVID.

* *p* < .05, ** *p* < .01, *** *p* < .001

### Supplemental Table 2. Association between Mood Disorder Status and EMA avolition, anhedonia, and asociality

| Negative Symptom | Mood Disorder Current | Mood Disorder Lifetime |
| --- | --- | --- |
| Avolition | .16 | .04 |
| Anhedonia | .12 | -.13 |
| Asociality | .12 | .14 |

Note = Mood disorder is scored such that 1 = presence of mood disorder and 0 = no mood disorder.

### Supplemental Table 3. Overall Negative Symptoms omnibus effects

| Context | Total R^2^ | Fixed-effect R^2^ | Group | Context | Group X Context |
| --- | --- | --- | --- | --- | --- |
| Activity | 0.48 | 0.06 | 4.33* | 323.85*** | 18.44* |
| Location | 0.42 | 0.04 | 3.13 | 161.26*** | 22.98*** |
| Social partner | 0.47 | 0.12 | 1.22 | 517.68*** | 19.17** |
| Social modality | 0.44 | 0.09 | 0.87 | 351.5*** | 18.12*** |

### Supplemental Table 4. Anticipatory and consummatory pleasure omnibus models

| Context | R2.total | R2.fixed | Group | Context | Group X Context |
| --- | --- | --- | --- | --- | --- |
| Anticipatory | | | | | |
| Activity | 0.44 | 0.1 | 12.28*** | 503.39*** | 22.1** |
| Location | 0.36 | 0.05 | 6.00* | 146.8*** | 9.84* |
| Social partner | 0.35 | 0.03 | 2.64 | 72.34*** | 5.29 |
| Social modality | 0.38 | 0.06 | 3.41 | 212.79*** | 9.91 |
| Consummatory | | | | | |
| Activity | 0.42 | 0.09 | 18.95*** | 468.67*** | 24.38*** |
| Location | 0.33 | 0.05 | 7.71** | 135.48*** | 12.79** |
| Social partner | 0.32 | 0.03 | 4.76* | 61.72*** | 5.86 |
| Social modality | 0.36 | 0.06 | 5.73* | 181.64*** | 6.94 |

### Supplemental Table 5. Omnibus model effects for Goal-Directed and Hedonic Activity Composites

| Context | Total R^2^ | Fixed-effect R^2^ | Group | Context | Group X Context |
| --- | --- | --- | --- | --- | --- |
| Anhedonia | | | | | |
| Goal-directed | 0.38 | 0.12 | 17.88*** | 386.3*** | 12.13*** |
| Hedonic | 0.37 | 0.11 | 2.49 | 369.88*** | 9.44** |
| Avolition | | | | | |
| Goal-directed | 0.3 | 0.04 | 12.61*** | 116.41*** | 7.39** |
| Hedonic | 0.33 | 0.08 | 4.11* | 234.51*** | 1.9 |
| Asociality | | | | | |
| Goal-directed | 0.38 | 0 | 0.29 | 3.11 | 0.06 |
| Hedonic | 0.38 | 0.01 | 1.15 | 11.46*** | 1.3 |
| Overall Negative Symptoms | | | | | |
| Goal-directed | 0.4 | 0.05 | 8.64** | 148.7*** | 5.66* |
| Hedonic | 0.42 | 0.08 | 3.59 | 251.67*** | 1.17 |

### Supplemental Table 6. Effects of preceding negative symptoms on context probability

| Context | R2 Total | R2 Fixed | Negative Symptoms | Group X Negative Symptoms | CHR slope | CN slope |
| --- | --- | --- | --- | --- | --- | --- |
| Activity |  |  |  |  |  |  |
| Goal-directed | 0.3 | 0.02 | 0.24***  [0.12, 0.36] | -0.21**  [-0.37, -0.06] | 0.03  [-0.08, 0.13] | 0.24  [0.12, 0.36] |
| Hedonic | 0.29 | 0.01 | -0.28***  [-0.4, -0.16] | 0.15  [-0.01, 0.31] | -0.13  [-0.24, -0.03] | -0.28  [-0.4, -0.16] |
| Resting | 0.32 | 0.02 | -0.11  [-0.29, 0.06] | 0.1  [-0.12, 0.32] | -0.01  [-0.14, 0.12] | -0.11  [-0.29, 0.06] |
| Studying/ Working | 0.43 | 0.02 | 0.23***  [0.09, 0.37] | -0.1  [-0.28, 0.08] | 0.13  [0.01, 0.24] | 0.23  [0.1, 0.36] |
| TV | 0.53 | 0.02 | -0.01  [-0.22, 0.2] | -0.11  [-0.37, 0.15] | -0.12  [-0.27, 0.04] | -0.01  [-0.22, 0.2] |
| Eating | 0.25 | 0.01 | -0.21**  [-0.35, -0.07] | 0.18*  [0, 0.36] | -0.03  [-0.15, 0.09] | -0.21  [-0.34, -0.07] |
| Computer | 0.43 | 0.01 | -0.07  [-0.22, 0.08] | 0  [-0.19, 0.18] | -0.07  [-0.19, 0.04] | -0.07  [-0.22, 0.08] |
| Errands | 0.45 | 0 | 0.18  [-0.02, 0.38] | -0.18  [-0.45, 0.08] | 0  [-0.18, 0.17] | 0.18  [-0.02, 0.38] |
| Recreation | 0.33 | 0.01 | -0.25**  [-0.41, -0.09] | 0.1  [-0.11, 0.31] | -0.15  [-0.29, -0.01] | -0.25  [-0.41, -0.09] |
| Commuting | 0.42 | 0.02 | 0.13  [-0.09, 0.34] | -0.3*  [-0.58, -0.02] | -0.17  [-0.34, 0] | 0.13  [-0.09, 0.34] |
| Location |  |  |  |  |  |  |
| Home | 0.51 | 0.03 | 0.04  [-0.09, 0.17] | -0.14  [-0.32, 0.04] | -0.1  [-0.22, 0.02] | 0.04  [-0.09, 0.17] |
| School/ Work | 0.66 | 0.02 | 0.35***  [0.17, 0.53] | -0.07  [-0.31, 0.16] | 0.28  [0.13, 0.43] | 0.35  [0.17, 0.53] |
| Public | 0.41 | 0.03 | -0.15  [-0.3, 0] | 0.1  [-0.11, 0.3] | -0.05  [-0.19, 0.08] | -0.15  [-0.3, 0] |
| Friend or family home | 0.92 | 0 | -0.44**  [-0.75, -0.13] | 0.57**  [0.18, 0.96] | 0.13  [-0.11, 0.37] | -0.44  [-0.75, -0.13] |
| Social Partner |  |  |  |  |  |  |
| No one/ Alone | 0.45 | 0.02 | 0.23***  [0.11, 0.36] | -0.07  [-0.24, 0.1] | 0.16  [0.05, 0.27] | 0.23  [0.11, 0.36] |
| Family | 0.49 | 0.01 | -0.13  [-0.28, 0.02] | -0.06  [-0.26, 0.15] | -0.19  [-0.33, -0.05] | -0.13  [-0.28, 0.02] |
| Significant other | 0.84 | 0.02 | -0.56***  [-0.81, -0.31] | 0.15  [-0.16, 0.47] | -0.4  [-0.6, -0.21] | -0.56  [-0.81, -0.31] |
| Classmates/ Coworkers | 0.64 | 0.03 | 0.24*  [0.02, 0.46] | -0.1  [-0.38, 0.18] | 0.14  [-0.04, 0.32] | 0.24  [0.02, 0.46] |
| Friends | 0.5 | 0.04 | -0.37***  [-0.53, -0.21] | 0.2  [0, 0.41] | -0.17  [-0.3, -0.03] | -0.37  [-0.53, -0.21] |
| Strangers | 0.84 | 0.01 | -0.03  [-0.34, 0.27] | -0.03  [-0.41, 0.36] | -0.06  [-0.29, 0.18] | -0.03  [-0.34, 0.27] |
| Social Modality |  |  |  |  |  |  |
| In-person | 0.43 | 0.03 | -0.25***  [-0.37, -0.12] | 0.04  [-0.12, 0.21] | -0.2  [-0.32, -0.09] | -0.25  [-0.37, -0.12] |
| Electronic (text, social media, etc) | 0.64 | 0.03 | 0.05  [-0.22, 0.31] | -0.17  [-0.49, 0.15] | -0.12  [-0.31, 0.07] | 0.05  [-0.22, 0.31] |
| Phone/ Video call | 0.5 | 0.03 | 0.15  [-0.13, 0.43] | -0.13  [-0.46, 0.2] | 0.02  [-0.17, 0.2] | 0.15  [-0.13, 0.43] |

* *p* < .05, ** *p* < .01, *** *p* < .001

## Figures

### Supplemental Figure 1. Overall Negative Symptoms


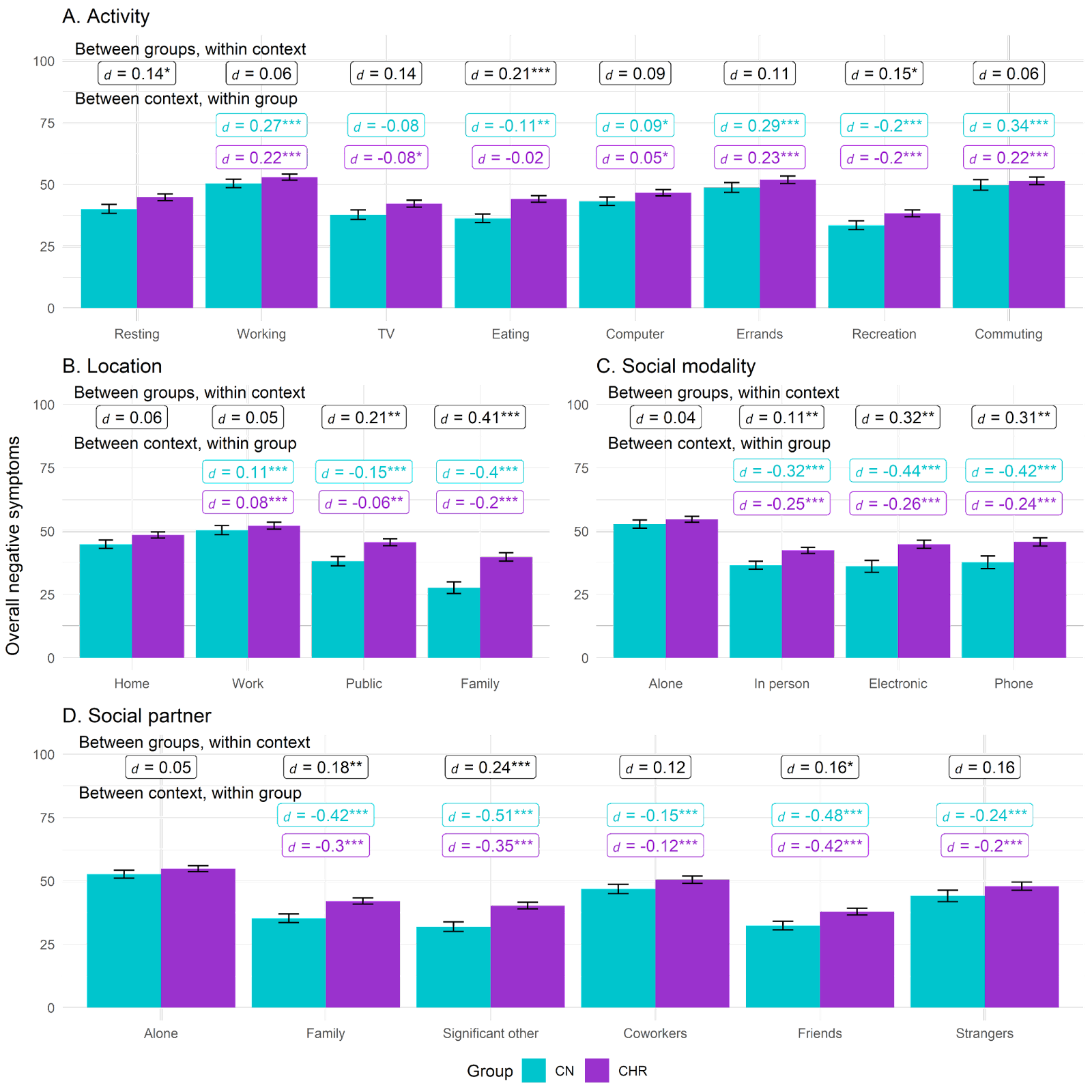


*Note.* Between groups, within context labels reflect contrast between groups within each context while between context, within group labels reflect contrast within group relative to reference context (e.g., resting or being at home). Figures use estimated marginal means and error bars reflect standard error. Work contexts also included school (e.g., item was school/work), while family location also includes friends.

* = *p* < .05, ** = *p* < .01, ** = *p* < .001

### Supplemental Figure 2. Anticipatory and consummatory pleasure - Activity


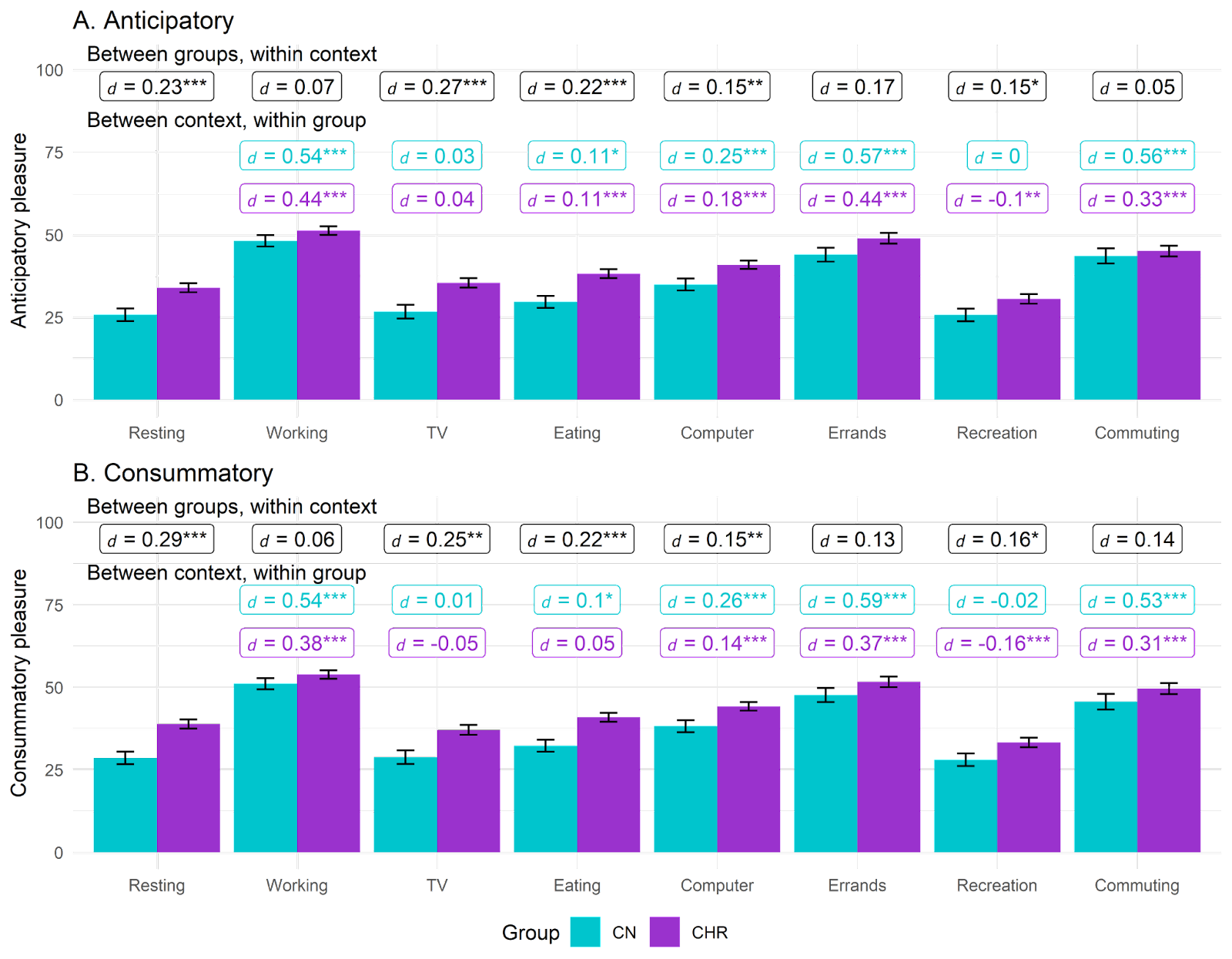


*Note.* Between groups, within context labels reflect contrast between groups within each context while between context, within group labels reflect contrast within group relative to reference context (e.g., resting or being at home). Figures use estimated marginal means and error bars reflect standard error. Work contexts also included school (e.g., item was school/work), while family location also includes friends.

* = *p* < .05, ** = *p* < .01, ** = *p* < .001

### Supplemental Figure 3. Anticipatory and consummatory pleasure - Location


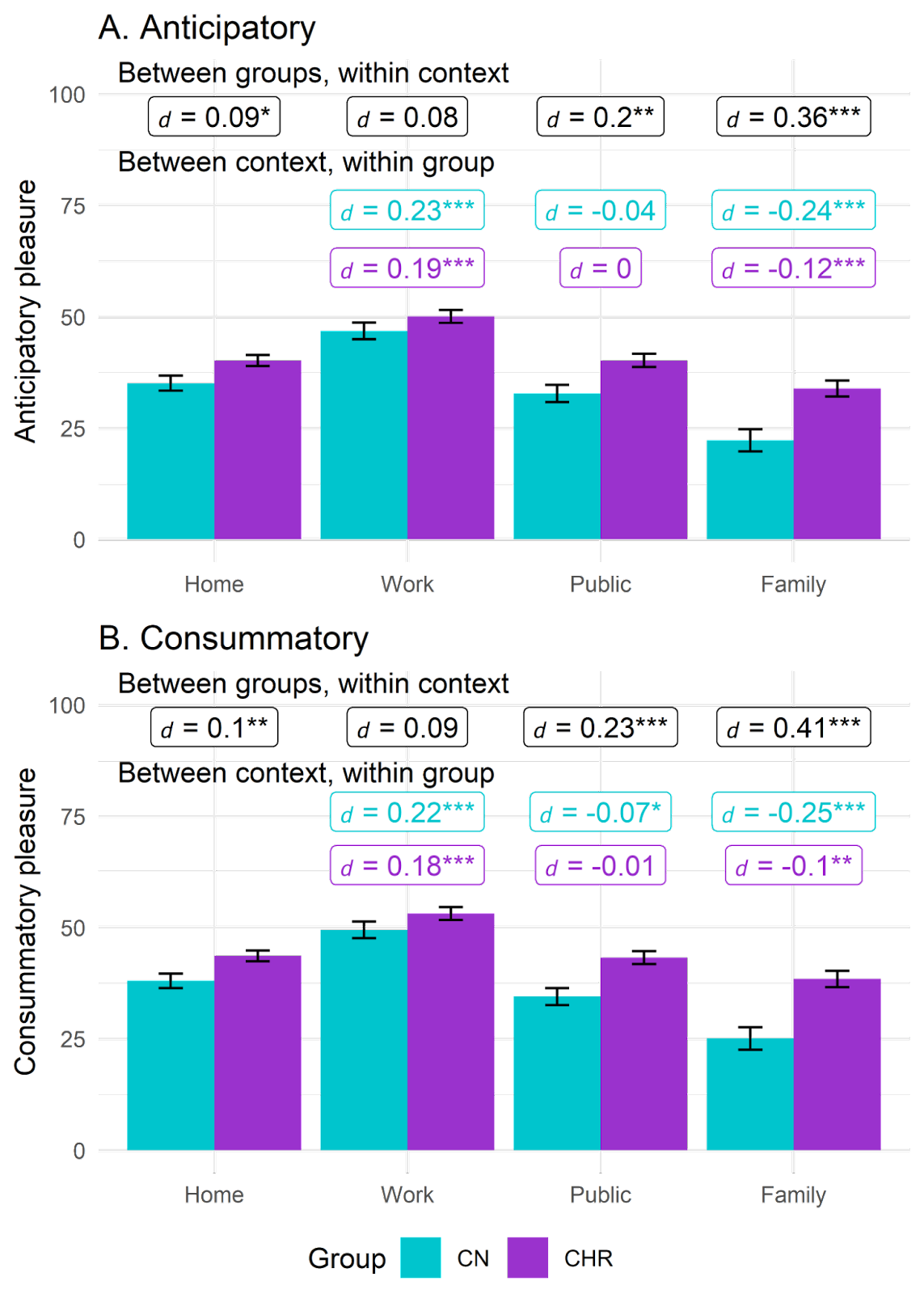


*Note.* Between groups, within context labels reflect contrast between groups within each context while between context, within group labels reflect contrast within group relative to reference context (e.g., being at home). Figures use estimated marginal means and error bars reflect standard error. Work contexts also included school (e.g., item was school/work), while family location also includes friends.

* = *p* < .05, ** = *p* < .01, ** = *p* < .001

### Supplemental Figure 4. Anticipatory and consummatory pleasure - Social


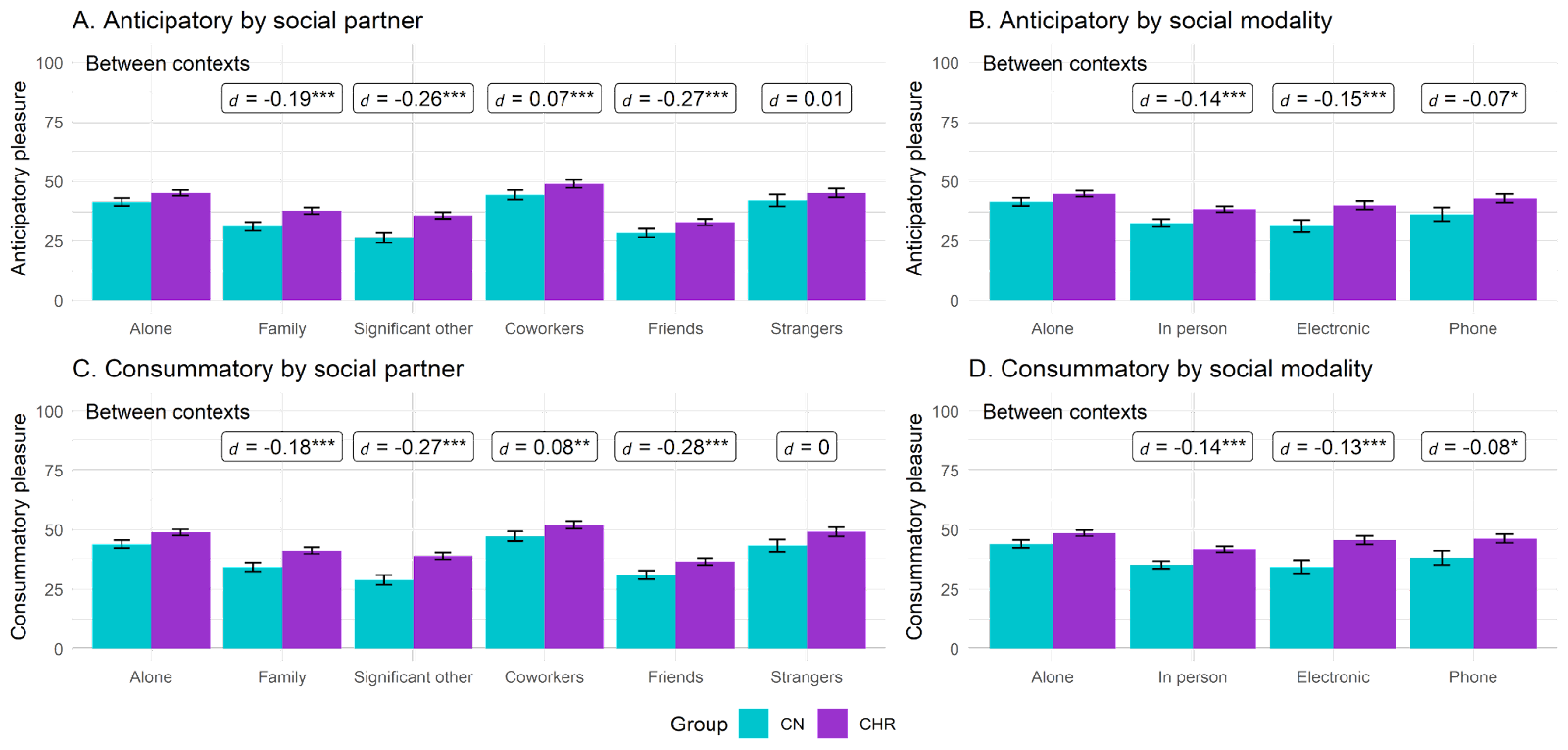


*Note.* Between groups, within context labels reflect contrast between groups within each context while between context, within group labels reflect contrast within group relative to reference context (e.g., resting or being at home). Figures use estimated marginal means and error bars reflect standard error. Work contexts also included school (e.g., item was school/work), while family location also includes friends.

* = *p* < .05, ** = *p* < .01, ** = *p* < .001

### Supplemental Figure 5. Hedonic and Goal-Directed Activities – Anhedonia


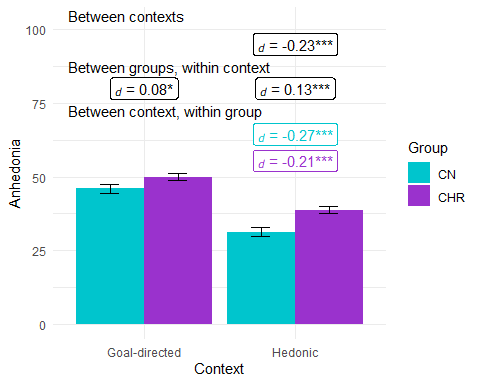


*Note.* Figures use estimated marginal means and error bars reflect standard error.

* = *p* < .05, ** = *p* < .01, ** = *p* < .001

### Supplemental Figure 6. Hedonic and Goal-Directed Activities – Avolition


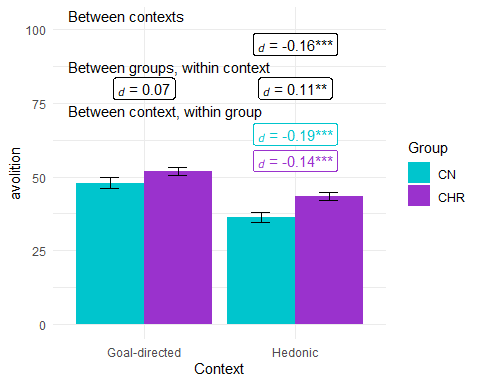


*Note.* Figures use estimated marginal means and error bars reflect standard error.

* = *p* < .05, ** = *p* < .01, ** = *p* < .001

### Supplemental Figure 7. Hedonic and Goal-Directed Activities – Asociality


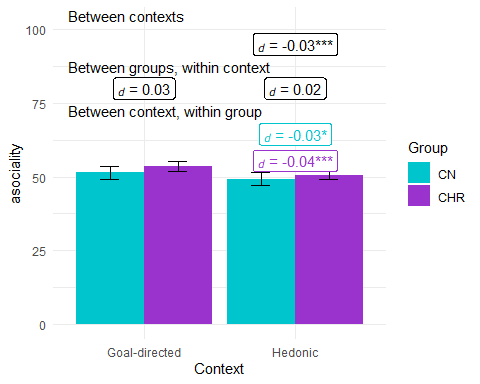


*Note.* Figures use estimated marginal means and error bars reflect standard error.

* = *p* < .05, ** = *p* < .01, ** = *p* < .001

### Supplemental Figure 8. Hedonic and Goal-Directed Activities – Overall Negative Symptoms


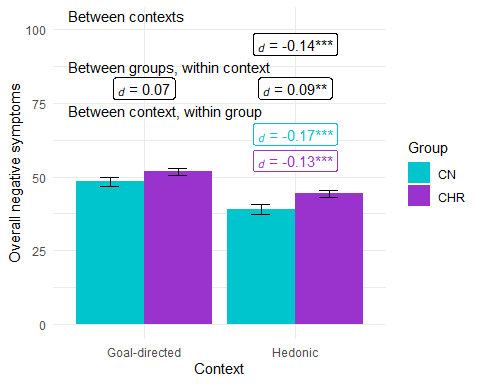


*Note.* Figures use estimated marginal means and error bars reflect standard error.

* = *p* < .05, ** = *p* < .01, ** = *p* < .001

## References

Murayama, K., Usami, S., & Sakaki, M. (2022). Summary-statistics-based power analysis: A

new and practical method to determine sample size for mixed-effects modeling.

*Psychological Methods*, 27(6), 1014–1038. https://doi.org/10.1037/met0000330
